# Six months of high-dose xylitol in high-risk caries subjects—a 2-year randomised, clinical trial

**DOI:** 10.1007/s00784-012-0774-5

**Published:** 2012-07-13

**Authors:** Guglielmo Campus, Maria Grazia Cagetti, Silvana Sale, Massimo Petruzzi, Giuliana Solinas, Laura Strohmenger, Peter Lingström

**Affiliations:** 1 Department of Surgery, Microsurgery, Medical Sciences, Dental School, University of Sassari, Viale San Pietro 43/C, 07100 Sassari, Italy; 2 WHO Collaborating Centre of Milan for Epidemiology and Community Dentistry, University of Milan, Milan, Italy; 3 Department of Dentistry and Surgery, University of Bari “Aldo Moro”, Bari, Italy; 4 Department of Biomedical Sciences, University of Sassari, Sassari, Italy; 5 Department of Cariology, Institute of Odontology, The Sahlgrenska Academy, University of Gothenburg, Gothenburg, Sweden

**Keywords:** Caries, Xylitol, Chewing gum, School-based, Randomised, controlled, clinical trial

## Abstract

**Objectives:**

The hypothesis was that the daily use of a high dose of a xylitol chewing gum for 6 months would reduce the increment of decayed permanent first molar surfaces (ΔD_6_S) in high-risk schoolchildren after 2 years.

**Methods:**

In this randomised, clinical trial, 204 schoolchildren with a high caries risk were assigned to two experimental groups, xylitol and non-xylitol. Caries status, salivary mutans streptococci, and lactobacilli were re-evaluated 2 years later in 74 xylitol-treated and 83 non-xylitol-treated schoolchildren. Differences in mean ∆D_6_S between groups registered at baseline and at follow-up were evaluated using the nonparametric Mann–Whitney *U* test.

**Results:**

Outcome was the development of detectable carious lesions initial (D_1_–D_2_) and manifest (D_3_) in the permanent first molars. In the xylitol group, the difference in proportion of children with decayed first permanent molars at baseline and follow-up was 1.43 % for manifest lesion and 2.86 % for initial lesions; while in the non-xylitol group was 10.26 % (*p* < 0.01) and 16.66 % (*p* < 0.01), respectively. A statistically significant difference regarding means was also observed in the non-xylitol group: the ∆D_6_S for manifest lesion was 0.18 (*p* = 0.03) and 0.67 (*p* = 0.02) for initial lesion.

**Conclusion:**

The use of a chewing gum containing a high dose of xylitol for a period of 6 months has been shown to produce a long-term effect on caries development in high caries-risk children.

**Clinical relevance:**

A school-based preventive programme based on 6 months’ administration of a high dose of xylitol via chewing gum proved to be efficacious in controlling caries increment in high-risk children.

## Introduction

Modern concepts regard caries as an interaction between genetic and environmental factors, where biological, social, behavioural and psychological factors are expressed in a highly complex interactive manner with the dental biofilm as the key element [1]. Different preventive approaches have focused on the reduction of sugar intake and its replacement with non-fermentable sweeteners, such as polyols. Today, the most used polyols are sorbitol and xylitol [2], which are incorporated in several products, such as chewing gums.

Xylitol is considered to be non-fermentable by oral bacteria, and it inhibits the growth, metabolism and polysaccharide production of mutans streptococci (MS), showing both non-cariogenic and cariostatic properties [3, 4]. Although studies have demonstrated the efficacy of low doses of xylitol [5, 6], others have speculated that a relatively high daily dose [7–10], as well as high-frequency consumption [11], is needed to obtain therapeutic effects. The regular use of xylitol chewing gum has been found to increase salivary flow and enamel remineralisation, and to reduce plaque acidogenicity and the growth of salivary lactobacilli (Lb) [2, 11, 12].

In order to choose the most effective treatment over time and the most optimal strategy from a cost-effectiveness angle, studies evaluating the effect of xylitol regimens on caries prevention are scant [13, 14]. A greater preventive effect on teeth erupting during and immediately after xylitol consumption has been described when using xylitol for a long period of time [15].

We have previously reported the effect of a high dose (11.6 g) of xylitol, administered daily via a chewing gum for 6 months, on plaque pH and salivary MS in a sample of high caries-risk schoolchildren [10]. The children were randomly divided into two groups: xylitol and non-xylitol. Children in the xylitol group showed a statistically significant reduction in both plaque acidogenicity and MS concentration. After 6 months of xylitol use, no caries prevention strategy was pursued at community level but only at individual level, using a fluoridated toothpaste. The hypothesis tested in this study was that the daily use of a high dose of a xylitol chewing gum (11.6 g) for 6 months would reduce the increment of decayed tooth surfaces (∆D_6_S) in the permanent first molars. The aim of this study was therefore to evaluate the increment of decayed tooth surfaces (∆D_6_S) in the permanent first molars 2 years after the daily use for 6 months of a high-dose xylitol chewing gum.

## Materials and methods

### Study design

**Fig. 1:**
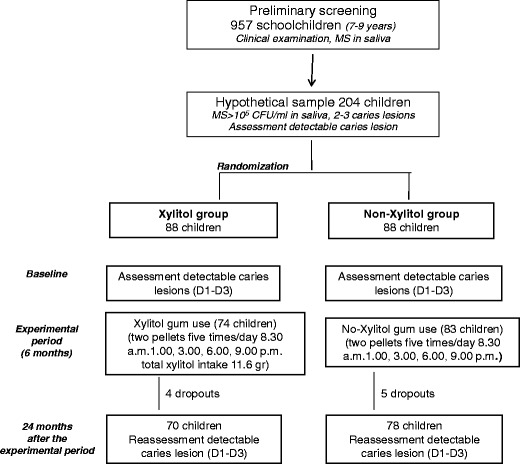
Flow chart of the study design

As previously reported [10], a randomised, clinical trial was carried out in Sassari (Italy). The survey was approved by the Ethics Committee at the Board of the Health office, University of Sassari (registration no. 2006/24). The fluoride concentration in the tap water in the area varies from 0.03 to 0.05 ppm [16]. An informed consent form to participate in the trial was given to parents or guardians, and only children with their parents’ signed consent were screened. Preliminary screening (*n* = 957) was carried out from October to December 2007 [16] to select children aged 7–9 years (mean age, 8.3 ± 1.2 years) presenting the following inclusion criteria: the presence of two or three carious lesions in the permanent and/or primary dentition, recorded at D_3_ level, a salivary MS concentration of >10^5^ CFU/mL and the presence of all the first permanent molars. Children with systemic disease or history of systemic antibiotic use during the 6 months before the beginning of the trial were excluded. A total of 231 subjects fulfilled the inclusion criteria. The power analysis of the RCT study was calculated considering a significant difference of 25 % between the test and the control group and a 95 % probability of obtaining a significant difference between groups at the 5 % level; the number of subjects per group was set at 66. Of the 231 subjects eligible in the study, 204 agreed to participate (acceptance rate, 88.3 %). The investigation was designed as a double-blind randomised, placebo-controlled study with two parallel arms and with an experimental period of 6 months. The subjects were compiled into a list, then using a computer program (Excel 2003 for Mac OsX), the randomization was carried out; two groups of children were created: a xylitol group, using a non-sucrose chewing gum containing xylitol, sorbitol, maltitol and mannitol, and a non-xylitol group, using a non-xylitol chewing gum where xylitol had been replaced by isomalt, an artificial sugar alcohol, made by a mixture of gluco-mannitol and gluco-sorbitol. The children were instructed to chew two pellets for 5 min, five times a day (8.30 a.m. and 1.00, 3.00, 6.00 and 9.00 p.m.), immediately after main meals and snacks supervised by teachers or parents. In all, 157 children completed the experimental period: 74 in the xylitol group and 83 in the non-xylitol group. In January–February 2010, 2 years after completing the chewing gum administration, the children were re-examined, and their caries status, salivary MS and Lb concentration were re-evaluated. The flow chart of the study design is shown in Fig. 1. During the experimental period, all the subjects received a fluoridated toothpaste containing 1,450 mg NaF (Mentadent P, Unilever Italy, Milan). During the interim period between the end of the experimental period and the follow-up examination, a questionnaire was submitted every 6 months in order to assess if the children had to undergo any caries preventive strategies. Diet regimen was also investigated. A 3-day diet diary was submitted together with the questionnaire. The mean daily sugar intake frequency and the total amount of sugar consumption were calculated. Sugar intake frequency was evaluated from the 3-day diet diary. The mean daily total amount was calculated summarising the sugar content (mono- and disaccharides) contained in all food and drink consumed for 3 days, obtained from a reference table [17]. No data on the socioeconomic status of the family and the dental attendance of the children were collected.

### Chewing gum

The xylitol chewing gum contained xylitol (36.6 %), sorbitol (17.7 %), maltitol (9.7 %), mannitol (7.1 %), gum base, flavours, humectants, food colour, acidity regulator and glazing agents. The ingredients of the non-xylitol chewing gum were isomalt (30.0 %), sorbitol (17.7 %), maltitol (16.3 %), mannitol (7.1 %), gum base, flavours, humectants, food colour, acidity regulator and glazing agents. Apart from sweeteners, the two chewing gums were identical in composition and in weight (3.17 g), shape, colour and packaging. They were produced and supplied by Perfetti Van Melle (Lainate, Italy). The total daily intake of xylitol was 11.6 g. The body’s tolerance of xylitol was assessed by means of a questionnaire administered to the participants’ parents shortly after the gum distribution had started and 3 months later, while the study was still proceeding. The questions focused on the potential side effects of using the gum. In order to evaluate the success of the administration of chewing gum at school and at home, teachers and parents were given chewing gums necessary for a single month at a time, and asked to return the empty blister packs when receiving those for the following month. This procedure was repeated for all 6 months of the experimental (chewing) period. Only three children did not return the empty blister, and so they were excluded from the trial.

### Caries registration

All the permanent first molars were evaluated for the presence of carious lesions. Carious lesions were scored by two blinded calibrated examiners, who worked in pair: one did the screening and the second collected data.

Both initial and manifest caries were scored (D_1_–D_3_ level) [18]. D_1_ was scored a clinically detectable enamel lesion without cavitation, D_2_ a cavity limited to enamel and finally D_3_ a cavity involving the dentine. Bitewing radiographs were taken and manually developed using standard conditions and standard processing times. The radiographs were finally examined on a backlit screen at ×2 magnification. The O’Mullane criteria were used to indicate enamel or dentine carious lesions [19].

Sealed molars were included in the survey and scored as sound; banded molars were also included and scored sound if the occlusal surface did not present caries lesions. The two examiners received training, and intra- and inter-examiner reliability was assessed before the beginning of the survey. Forty-five subjects were re-examined after 72 h by the two examiners. Inter-examiner reliability was evaluated through analysis of variance for fixed effect [20], while intra-examiner reproducibility was assessed as percentage agreement and Cohen’s kappa statistics. Good inter-examiner reliability (*p* = 0.21) with a low value of mean squares for error (0.44) was found. As regards intra-examiner reproducibility, the percentage agreement was high (Cohen’s kappa 0.84). When the same procedure was performed before the follow-up examinations, good inter-examiner reliability (*p* = 0.24) and a high percentage agreement (Cohen’s kappa 0.86) were found.

The D_6_FS index expressed the total prevalence of interproximal enamel and dentine caries, assessed by both radiographic and clinical evaluation, and surfaces with fillings for the first permanent molars. The net caries increment (∆D_6_S) was calculated as the difference between the follow-up and baseline scores [18].

At baseline and at the 2-year follow-up examinations, a stimulated whole saliva sample was collected for 150 s in sterile vials (Nunc, Kamstrup, Denmark), following the clinical examination in standardised conditions. Samples were collected between 8:30 and 10:30 a.m., and tooth brushing was not allowed in the morning, before the collection. Each child chewed a piece of sterile paraffin wax for 60 s, after which the sample was collected. Mutans streptococci and lactobacilli counts in saliva were assessed and categorised using the dip-slide technique (CTR bacteria, Ivoclar Vivadent, Germany), following the manufacturer’s instructions.

### Data analysis

Several deciduous teeth were exfoliated at the 2-year follow-up examination and, as a result, the net caries increment was only calculated on the first permanent molar (∆D_6_S). Differences between groups in terms of the mean ∆D_6_S registered at baseline and at follow-up were evaluated using the nonparametric Mann–Whitney *U* test. Differences in proportion relating to microbiological counts and proportion of decayed first permanent molars at baseline and follow-up were assessed using equality of proportion test. The efficacy and consequences of treatment were also considered, calculating the event rate (ER) for each group and the number needed to treat (NNT) [21]. An event was defined as the presence of a new carious lesion at surface level, developed during the 2-year follow-up period. The data were analysed using statistical software (STATA version 10.1, USA); *p* < 0.05 was considered statistically significant.

## Results

A total of 157 subjects completed the xylitol clinical, randomised, intervention trial. Of these, 148 had a follow-up caries assessment after 2 years and were included in the final analysis. There were nine dropout subjects (5.7 %), during the 2-year period. The reasons for dropping out were the change of school (three subjects) or moving out of the community (six subjects). No side effects were observed in any of the subjects.

None of the subjects underwent any professional topical fluoride or antibacterial agent application, sealants or professional tooth cleaning during the follow-up period. Dietary habits did not differ statistically in the two groups, or with regard to the mean total amount of daily sugar consumption, nor as regards the intake frequency. Moreover, the use of chewing gum was fairly insignificant; only 12 (8.1 %) subjects reported the regular use (once a day or more) of sugar-free chewing gum (data not in table). Salivary bacteria (MS and Lb) results are shown in Table 1. At baseline, all the subjects had MS levels of >10^5^ CFU/ml as an inclusion criterion for the trial. No statistically significant decrease in the percentage of subjects with an MS of >10^5^ CFU/ml was noted in the two groups (5.7 % in the xylitol group and 5.1 % in the non-xylitol group) at the follow-up examination. The percentage of subjects with Lb >10^6^ CFU/ml at baseline was similar in the two groups (range, 93.4–94.3 %), while a statistically significant decrease was observed at the 2-year examination both in the xylitol group (75.7 %, *p* < 0.01) and in the non-xylitol group (82.1 %, *p* = 0.03).

**Table 1:** Salivary concentration of mutans streptococci (MS) and lactobacilli (Lb)

	Xylitol group, *n* = 70		Non-xylitol group, *n* = 78	
	Salivary MS >10^5^ CFU/ml, *n* (%)	Salivary Lb >10^6^ CFU/ml, *n* (%)	Salivary MS >10^5^ CFU/ml, *n* (%)	Salivary Lb >10^6^ CFU/ml, *n* (%)
Baseline	70 (100.0)	66 (94.3)	78 (100.0)	73 (93.4)
Follow-up	66 (94.3)	53 (75.7)	74 (94.9)	64 (82.1)
*p* value^a^	0.06	<0.01	0.12	0.03

Differences in proportion evaluated using equality of proportion test

^a^Equality of proportion test

No permanent first molars were extracted during the 2-year follow-up period. The caries index registered at baseline and at the 2-year follow-up is shown in Table 2. No statistically significant differences between the groups were recorded at baseline. The difference in proportion of children with decayed first permanent molars at baseline and follow-up within the groups was evaluated both for initial (D_1_–D_2_) and manifest (D_3_) levels. In the xylitol group, the difference in proportion was 1.43 % for manifest lesion and 2.86 % for initial lesions; while in the non-xylitol group, the difference in proportion was 10.26 % (*p* < 0.01) for manifest lesions and 16.66 % (*p* < 0.01) for initial lesions. A statistically significant difference regarding means was also observed in the non-xylitol group: the ∆D_6_S at D_3_ was 0.18 (*p* = 0.03) and 0.67 (*p* = 0.02) at D_1_–D_2_.

**Table 2:** Baseline and 2-year follow-up caries index D_6_FS, registered at D_3_ and D_1_-D_3_ level

	Xylitol group, subjects = 0				Non-xylitol group, subjects = 78			
	Manifest lesion (D_3_)		Initial lesion (D_1_–D_2_)		Manifest lesion (D_3_)		Initial lesion (D_1_–D_2_)	
	D_6_FS >0 subjects (%)	D_6_FS mean (SE)	D_6_FS >0 subjects (%)	D_6_FS mean (SE)	D_6_FS >0 subjects (%)	D_6_FS mean (SE)	D_6_FS >0 subjects (%)	D_6_FS mean (SE)
Baseline	40 (57.14)	0.61 (0.17)	54 (77.14)	1.52 (0.18)	44 (56.41)	0.61 (0.15)	60 (76.92)	1.45 (0.18)
Follow-up	41 (58.57)	0.62 (0.19)	56 (80.00)	1.65 (0.17)	52 (66.67)	0.79 (0.17)	73 (93.59)	2.12 (0.20)
∆D_6_S value	1 (1.43)^a^	0.01 (0.01)^b^	2 (2.86)^b^	0.13 (0.04)^c^	8 (10.26)^c^	0.18 (0.05)^d^	13 (16.66)^c^	0.67 (0.06)^e^

Value expressed as caries prevalence, means and standard errors

^a^Equality of proportion test *p* = 0.10

^b^Nonparametric Mann–Whitney *U* test *p* = 0.12

^c^Equality of proportion test *p* < 0.01

^d^Nonparametric Mann–Whitney *U* test *p* = 0.03

^e^Nonparametric Mann–Whitney *U* test *p* = 0.02

A gender difference for ∆D_6_S was found in the xylitol group, with 0.48 in females vs 0.60 (*p* = 0.04) in males (data not in table).

The total number of healthy surfaces at baseline was 1,224 in the xylitol group and 1,447 in non-xylitol group. The ER was 0.006 and 0.036, in the two groups, respectively. The NNT was 33.23 for the non-xylitol group vs the xylitol group (Table 3). The relative risk reduction was 82.1 % on the xylitol group compared to the non-xylitol one.

**Table 3:** Efficacy of the treatment

	Events (E)	Non-events (N)	Event rate (ER)
Xylitol group	8	1,216	0.006
Non-xylitol group	53	1,394	0.036

An event was defined as a new carious lesion (initial or manifest), developed during the 2-year follow-up period in both groups; NNT = 33.23

## Discussion

Several studies have demonstrated the efficacy of xylitol in caries prevention [8–10, 12], although evidence of its full effect is still limited. To evaluate the real efficacy of the preventive measures, it is crucial to know whether the positive effect lasts over time, even after its interruption.

The hypothesis of this study was that the daily use of a high dose of a xylitol chewing gum (11.6 g) for 6 months would reduce the increment of decayed tooth surfaces (∆D_6_S) in the permanent first molars in high caries-risk children. The study focused both on the concentration of cariogenic bacteria in saliva and on the presence of active carious lesions in the first permanent molars. The caries evaluation follow-up was performed after 2 years, during which no other community-based caries prevention strategies were pursued in the sample. A low dropout rate was recorded during this time period. At the 2-year follow-up, statistically significant differences in caries increment were observed in the permanent first molars between the groups. Subjects using the xylitol chewing gum showed a significantly lower increment in the number of new carious lesions.

Several trials have demonstrated the short-term effect of the polyol on MS [3, 6, 7, 10, 11], but only a few of them have investigated the effect of xylitol on salivary Lb [4, 22]. The results showed that the bacterial counts (MS and Lb) remained constant, despite a reduction in plaque acidogenicity [4, 22].

A statistically significant decrease in the number of children with a high salivary Lb concentration was found in both groups. However, the effect of chewing gum use on salivary lactobacilli was less pronounced in the non-xylitol group. A possible hypothesis might be linked to the age of the subjects at the follow-up examination. The inclusion criterion for the trial was the presence of two to three carious lesions in the permanent and/or deciduous dentition, so it is reasonable to speculate that several decayed deciduous teeth were exfoliated at the 2-year follow-up examination, producing a decrease of Lb concentration in saliva.

A growth-reducing effect of xylitol on salivary MS has been described, suggesting a long-term outcome of the polyol [12]. In the present report, no significant findings were observed regarding the effects of xylitol on the concentration of MS, although the xylitol group showed a numerical decrease in subjects with a high salivary MS concentration. One possible reason for this finding could be that these bacteria strains are MS ‘xylitol resistant’ instead of MS ‘xylitol sensitive’, with less cariogenicity potential as a consequence [23–25].

A reduction in the sugar metabolism of the oral biofilm has previously been reported in the same group of children as in the present study after both 3 and 6 months’ use and 3 months after the end of xylitol use [10]. Even if a clear antimicrobial effect could be found, this finding demonstrates a prolonged effect of the xylitol on different plaque-related variables. This paper thus appears to confirm the efficacy of xylitol chewing gum in caries prevention [13–15].

A statistically significantly lower caries increment was detected in females belonging to the xylitol group. A similar gender difference has previously been described [13, 14], and the hypothesis used as support was the more regular use of xylitol gum by the girls. In the original study [10], no differences regarding gender were found in terms of cariogenic bacteria and plaque pH, as well as regular chewing gum use. However, it is important to underline that due to the high daily dose administered, the intervention was demanding. A possible explanation could be related to a better gingival health status found in girls compared to males, related to a better level of oral hygiene [25]. The oral hygiene indices were not evaluated in the present trial, and differences among children in oral hygiene maintenance could have influenced the outcome of the present study. Nevertheless, caries lesion development is not correlated to dental biofilm at all, but to acidogenic and aciduric bacteria contained in it when sugar frequency increased in diet [26]. The total amount and frequency of the daily sugar consumption through food and drink were reported to be similar in the two groups, and no statistical differences were found.

No data on the socioeconomic status of the family were collected. The association between low socioeconomic background and caries disease is extensively described; nevertheless, the impact of the socioeconomic status on caries development in the present sample was considered negligible because the most important behaviour risk factors for caries as fluoride use and sugar consumption in diet were periodically checked during the follow-up period, and they did not significantly differ between the two groups.

The effect of a short period of treatment proved to be effective, thereby making it possible to allocate resources to additional preventive strategies. This study focused on the effect on the first permanent molars. However, a similar power would be expected for other tooth groups, which would increase interest in an intervention of this kind.

A clinical suggestion deriving from the results of this trial is the opportunity to administer a high dose of xylitol daily for short intervals to high caries-risk children. Even if the results of this trial are promising, the follow-up examination was performed just after 2 years, so a new, longer-term follow-up evaluation is needed to build up evidence for this kind of preventive procedure.
